# Health systems, rehabilitation care and COVID-19: Challenges and opportunities

**DOI:** 10.3389/fresc.2023.1134461

**Published:** 2023-02-10

**Authors:** Carlotte Kiekens, Antony Duttine, Satish Mishra, Carla Sabariego

**Affiliations:** ^1^ Department of Physical and Rehabilitation Medicine, IRCCS MultiMedica, Milan, Italy; ^2^ Advisor, Disability and Rehabilitation, Pan American Health Organization/World Health Organization, Washington, DC, United States; ^3^ Technical Officer, Division of Country Health Policies and Systems, WHO Regional Office for Europe, Copenhagen, Denmark; ^4^ Department of Health Sciences and Medicine, University of Lucerne, Lucerne, Switzerland; ^5^ Swiss Paraplegic Research, Nottwil, Switzerland; ^6^ Center for Rehabilitation in Global Health Systems, WHO Collaborating Center, University of Lucerne, Lucerne, Switzerland

**Keywords:** COVID-19, health systems, disability, rehabilitation, pandemics

**Editorial on the Research Topic** Health systems, rehabilitation care and COVID-19: Challenges and opportunities

COVID-19 has considerably challenged health systems worldwide, especially acute and intensive care services, and has had a profound impact on the rehabilitation sector. COVID-19 has increased the need for rehabilitation services, either for those recovering from more severe cases potentially requiring hospitalization, prolonged intensive care, or experiencing Post Intensive Care Syndrome (PICS) (1), or in relation to the growing evidence of long-term consequences of the disease across the population (post COVID-19 condition) (2, 3). On the other hand, continuity of rehabilitation care for non-COVID-19 patients and service users has often been disrupted as they were considered as “non-essential”, causing a potentially detrimental effect on this population. The importance of rehabilitation for COVID-19 has been recently stressed in a Call for Action of the World Health Organization (WHO) Regional Office for Europe (4), which focuses on the adoption of integrated care models to manage the post Covid-19 condition. In this call, two of four recommendations refer to rehabilitation, highlighting the need for individualized long-term rehabilitation as well as the need for strengthening health systems to provide it in a systematic and evidence-informed manner. Considering the opportunity to “build back better” after the COVID-19 pandemic, it is important to learn how rehabilitation services, provided through health systems, are responding to the needs of patients with and without COVID-19 across all phases of the continuum of care. Therefore, we launched this Research Topic on research that addresses how health systems across the world are responding to rehabilitation care needs during the COVID-19 pandemic, for COVID-19 and non-COVID-19 patients needing rehabilitation.

In this special collection, we are publishing four papers. Two papers are dealing with the impact of the COVID-19 pandemic on rehabilitation services: one tackles the countries' response to persons with disabilities (PWD) Lugo-Agudelo et al. while the second describes the impact and challenges in Bangladesh Uddin et al. A third paper presents a protocol for a study on rehabilitation and return-to-work of professionals who acquired COVID-19 in the workplace Muller et al. The fourth paper reports the results of an interdisciplinary workshop on the challenges of conducting rehabilitation research during the COVID-19 pandemic, and approaches to address the related complexities were proposed Noguchi et al.

Lugo-Agudelo et al. performed a broad search on measures adopted for PWD, among others, for containment, mitigation or suppression of the SARS-CoV-2 virus Lugo-Agudelo et al. They found information for 98 countries, with official documents only in 32 countries. The search focussed on all measures and was not specific to rehabilitation, though the authors noted that PWD may be an important rehabilitation user group. Indeed, the WHO, as well as the Centers for Disease Control and Prevention both declared PWD as more likely to become infected or develop serious illness (5). The identified documents mostly contained information and recommendations on the prevention of contagion and how to deal with the situation while at home or in isolation, or guidance for institutions for PWD. Social and economic benefits for PWD during the pandemic were as well reported. Lastly, the authors described specific measures for target groups with different types of impairments. Little information was found though on continuity of care for people who could benefit of rehabilitation, and for the prevention and response to violence, in particular concerning women with disabilities.

Uddin and colleagues' paper on COVID-19 pandemic in Bangladesh further illustrates the above-mentioned issues in a low-income country Uddin et al. The challenges are huge, starting with a very low vaccination rate. The healthcare budget is very low with <1% of the gross domestic product and two-thirds of the total health expenditure is out-of-pocket. There is an important shortage of health professionals, in particular regarding the rehabilitation workforce. During the pandemic, they were deployed in COVID-19 services and a high number of physicians and nurses got infected, of which some died. Also in Bangladesh PWD encountered difficulties with supplies and treatment. The authors express the need to upscale rehabilitation services in all phases of care: acute, post-acute, and long-term for PWD as well as for general population patients during and after COVID-19.

In high-income countries, such as Germany, health professionals were also infected with the SARS-CoV-2 virus. Muller and her team developed an interesting study protocol for an observational cohort study on patients who acquired COVID-19 in the workplace Muller et al. They present a very complete multimodal and interdisciplinary inpatient rehabilitation programme with a minimum duration of 3 weeks. The program includes psychological therapy, sports therapy, physiotherapy, respiratory therapy, and health promotion interventions, and starts in the post-acute phase of the disease. Extensive functional assessments of physical capacity, mental health and work ability at four points in time, up to 12 months after the start of the programme, shall shed light on the long-term effects of rehabilitation on COVID-19 long-term symptoms, in all its degrees of severity.

Noguchi and a team of collaborators organized a two-hour online workshop attended by 25 rehabilitation researchers to discuss the challenges they had experienced and strategies employed to conduct rehabilitation research during the COVID-19 pandemic Noguchi et al. The main topics were (1) pandemic protocol adjustments to public health guidelines; (2) participant accessibility to ensure equity, safety, and feasibility; (3) adapted knowledge dissemination such as smaller networking events, and revisiting timeframes.

This collection contributes to the knowledge about the impact and challenges of COVID-19 on rehabilitation services in health systems across the world, non-pandemic related rehabilitation research and introduces an observational study that will examine the effect of a comprehensive rehabilitation programme for patients who got infected with the SARS-CoV-2 virus at work, also on the long-term.

We are convinced that a “new” disease like COVID-19, for which evidence on many aspects is being continuously generated and compiled (6), requires rehabilitation academics and practitioners to share lessons learned in different settings. Lessons learned span from setting up sound research to collect data on the course of the disease and the effect of interventions provided to taking measures to ensure that rehabilitation services for non-infected patients are guaranteed. This research topic facilitates sharing experiences, from a health systems perspective, and reiterates that rehabilitation is not only relevant for chronic health conditions but also for infectious diseases like COVID-19, which can lead to important and long-lasting losses in functioning.
